# Another Dengue Antiviral Drug Bites the Dust Early: Where Does Dengue Therapeutic Drug Development Go from Here?

**DOI:** 10.4269/ajtmh.25-0362

**Published:** 2025-07-10

**Authors:** Swee Sen Kwek, Jenny G. Low

**Affiliations:** ^1^ Department of Infectious Diseases, Singapore General Hospital, Singapore;; ^2^ Programme in Emerging Infectious Diseases, Duke-National University of Singapore Medical School, Singapore;; ^3^ Viral Research and Experimental Medicine Centre, SingHealth-Duke National University of Singapore Academic Medical Centre, Singapore

Dengue virus (DENV) is a mosquito-borne orthoflavivirus that circulates as four serotypes (DENV1-4) and causes an acute illness that ranges from a mild febrile illness to more severe dengue, whereby life-threatening shock with severe haemorrhagic complications and multi-organ failure ensue if not appropriately managed.^1^ Current control strategies targeting the *Aedes aegypti* mosquito vector include insecticides, environmental controls^2^, and the release of Wolbachia, an endosymbiont of infected mosquitoes, to reduce the mosquito population and prevent carriage of dengue viruses.^3^^,^^4^ After decades of development efforts, three vaccines have been licensed for the prevention of dengue, although Sanofi Pasteur announced stopping production of the first generation vaccine, Dengvaxia®, in 2024, due to low global demand.^5^ Of note, none of the licensed vaccines demonstrated complete protection against symptomatic infection from all four DENVs.^6^^-^^10^ Several other vaccine candidates are in various stages of clinical development.^11^

While much progress have been made to control and prevent dengue, the geographic distribution of both DENVs and *Aedes* vectors continues to spread, fuelled by uncoordinated urbanization, global travel, and climate change.^12^^,^^13^ Half of the world’s population is at risk for dengue infection as both virus and vector spread beyond tropical regions.^12^ Dengue drug development thus remains critical as part of an overall control strategy and to meet an urgent need to reduce morbidity and mortality from severe disease. Dengue pathogenesis is driven by viral replication leading to viral-mediated cell death, but also by a cytokine and inflammatory cascade that drives plasma leakage, a hallmark of severe dengue.^1^ As such, dengue drug development can be approached from 2 fronts: 1) inhibition of DENV replication using direct-acting antivirals or inhibitors of host factors essential for viral replication; and 2) inhibition of host responses to infection that contribute to plasma leakage.^14^ Research and development have mainly focused on targeting the virus, as both lower viremia at diagnosis and shorter duration of viremia have been associated with reduced risk of severe dengue.^15^^,^^16^ However, treatment would likely need to be started very early after symptom development. As a result, dengue drug trials have included recruitment within the first 48 hours of symptom onset, with the assumption that antivirals that reduce viremia will reduce severity and improve disease outcomes.^14^

In this issue of the *American Journal of Tropical Medicine & Hygiene*, Arantxa Horga et al.^17^ report results of a Phase 2 randomized, double-blind placebo-controlled study to evaluate the efficacy and safety of AT-752 in NS1-positive confirmed dengue infection. AT-752 is an oral, highly potent, pan-serotype guanosine nucleotide DENV polymerase inhibitor that is well-tolerated at dosing of 750 mg three times daily.^18^ Direct-acting antivirals against DENV need to reach therapeutic serum levels quickly so as to rapidly halt viral replication, as demonstrated in a Phase 1 study of AT-752, with the drug metabolites achieving peak serum levels within an hour of ingestion and remaining detectable over 6 hours.^18^

In this phase 2 trial, enrollment of 60 dengue patients in 3 cohorts from 3 countries across 7 study sites was planned. However, the study was prematurely terminated after just 21 patients were recruited. The trial primary endpoint was the change in dengue viremia from baseline, but at interim, it was found that 23% in the treatment group and 43% in the placebo group had viremias below the limit of detection at baseline. In March of 2023 the trial was terminated due to logistical challenges and escalating costs associated with having to enroll sufficient patients to account for marked variability in viremia levels.^19^ Consequently, the trial primary endpoint was unevaluable. Post-hoc analysis nonetheless showed that the treatment group had greater reduction in viremia amongst those with detectable levels at baseline. The time to sustained fever resolution was 4 days in the treated group compared to >5 days in the placebo group. Platelet recovery similarly appeared to be faster amongst patients who received AT-752, though the sample size was small for this comparison. The drug was generally well tolerated, and its PK profile was consistent with phase 1 findings.

The early retirement of this trial illustrated the challenges of antiviral dengue drug studies. One key challenge is the narrow window for patient recruitment given that the febrile phase of dengue is non-specific and undistinguishable from that of other viral illnesses. Subjective reporting of fever by patients is also known to be highly unreliable, and dengue is best distinguished from other febrile illnesses through its symptomology and laboratory findings later in the course of infection.^1^^,^^20^^,^^21^ Unsurprisingly, although this trial was intended to enroll patients with early dengue within 48 hours of fever onset, viremia was below the limit of detection in 30% of patients at recruitment and was in rapid decline by Day 4 of illness. Another challenge is the limited sensitivity of available diagnostic tools for dengue.^22^^,^^23^ Recruitment of trial patients has relied on the point-of-care DENV sNS1 test, but assay sensitivity is low in the first two days after fever onset and in secondary dengue.^24^ Dengue PCR is more sensitive, but screening of acutely febrile patients with this assay would not be practical in most clinical settings. Therefore, despite the unmet need, the practical challenges of direct-acting antiviral therapeutic trials are unlikely to be resolved easily.

There are nonetheless reasons to stay optimistic. Advances in the understanding of dengue pathogenesis have been made in the last 2 decades. It is now known that the systemic inflammatory response to infection is an important contributor to endothelial dysfunction and plasma leakage. Importantly, plasma concentrations of pro-inflammatory cytokines have been found to track with the magnitude of viremia and to persist in patients with severe dengue even after viremia resolves.^15^^,^^16^^,^^25^ These findings suggest that targeting the dysregulated host immune response using immunomodulatory drugs offers a wider window of opportunity for treatment than direct-acting antivirals. Indeed, corticosteroids, immunoglobulins, and other repurposed drugs to target different immune and metabolic responses to dengue have been investigated or are being studied To date, corticosteroids and immunoglobulins have not been shown to help, but they are non-specific immunosuppressants.^26^^,^^27^ Whether there is a key pathogenic immune pathway driving severe dengue that can be druggable to attenuate the implicated cytokine(s) should to be urgently investigated. Finally, more sensitive yet affordable point-of-care diagnostic tools to identify dengue early could be developed in the near future^28^ to expedite early treatment.

Dengue is here to stay. It will remain a major global health threat in the years to come.^12^ A multi-pronged approach involving vector control, primary vaccination, and therapeutics is a must in limiting severe illness and mortality. Even as AT-752 has bitten the dust early and another agent, JNJ-1802,^29^ was shelved for similar reasons,^30^ there is extensive ongoing academic effort to gain deeper understanding of dengue pathogenesis for discovery of therapeutic targets and better vaccines. It is therefore essential to build on the current momentum for sustainable collaborations and strengthen partnerships between academia, industry, governments, and non-governmental bodies to achieve the goal of zero dengue deaths.
